# Defining the scope of extended NIPS in Western China: evidence from a large cohort of fetuses with normal ultrasound scans

**DOI:** 10.1186/s12884-023-05921-x

**Published:** 2023-08-19

**Authors:** Lin Chen, Li Wang, Yang Zeng, Daishu Yin, Feng Tang, Dan Xie, Hongmei Zhu, Hongqian Liu, Jing Wang

**Affiliations:** 1 Department of Medical Genetics, West China Second University Hospital, Sichuan University, Chengdu, 610041 China; 2 Key Laboratory of Birth Defects and Related Diseases of Women and Children, Sichuan University, Ministry of Education, Chengdu, 610041 China; 3 Department of Obstetrics and Gynecology, West China Second University Hospital of Sichuan University, Block 3 No. 20, Ren Min Nan Road, Wuhou district, 610041 Chengdu, China

**Keywords:** Noninvasive prenatal screening (NIPS), Extended noninvasive prenatal screening, Pathogenic/likely pathogenic copy number variants (P/LP CNVs), Fetus, Invasive prenatal diagnosis

## Abstract

**Background:**

Standard noninvasive prenatal screening(NIPS) is an accurate and reliable method to screen for common chromosome aneuploidies, such as trisomy 21, 18 and 13. Extended NIPS has been used in clinic for not only aneuploidies but also copy number variants(CNVs). Here we aim to define the range of chromosomal abnormalities that should be able to identify by NIPS in order to be an efficient extended screening test for chromosomal abnormalities.

**Methods:**

A prospective study was conducted, involving pregnant women without fetal sonographic structural abnormalities who underwent amniocentesis. Prenatal samples were analyzed using copy number variation sequencing(CNV-seq) to identify fetal chromosomal abnormalities.

**Results:**

Of 28,469 pregnancies included 1,022 (3.59%) were identified with clinically significant fetal chromosome abnormalities, including 587 aneuploidies (2.06%) and 435 (1.53%) pathogenic (P) / likely pathogenic (LP) CNVs. P/LP CNVs were found in all chromosomes, but the distribution was not uniform. Among them, P/LP CNVs in chromosomes 16, 22, and X exhibited the highest frequencies. In addition, P/LP CNVs were most common on distal ends of the chromosomes and in low copy repeat regions. Recurrent microdeletion/microduplication syndromes (MMS) accounted for 40.69% of total P/LP CNVs. The size of most P/LP CNVs (77.47%) was < 3 Mb.

**Conclusions:**

In addition to aneuploidies, the scope of extended NIPS should include the currently known P/LP CNVs, especially the regions with recurrent MMS loci, distal ends of the chromosomes, and low copy repeat regions. To be effective detection should include CNVs of < 3 Mb. Meanwhile, sufficient preclinical validation is still needed to ensure the clinical effect of extended NIPS.

**Supplementary Information:**

The online version contains supplementary material available at 10.1186/s12884-023-05921-x.

## Background

Chromosomal abnormalities are the most common genetic etiology of birth defects. Therefore, every pregnant woman should be offered the choice of early screening for chromosomal abnormalities [1, 2]. The fetal chromosomal abnormalities mainly include aneuploidies and unbalanced chromosomal rearrangements, which include copy number variants (CNVs). Unlike the incidence of aneuploidy which increases with maternal age, the incidence of CNVs is independent of maternal age [3–5]. For patients of any age with a normal ultrasound and karyotype, the chance of carrying pathogenic(P)/likely pathogenic (LP) CNVs is greater than 1%, similar to the age-related risk of aneuploidy in the fetus of a 38 year old pregnant woman [6, 7]. Array comparative genomic hybridization (aCGH), first proposed in 1997, has served as a robust and effective approach to screen for CNVs [8]. In recent years, CNV analysis based on next generation sequencing (NGS) technology has been widely applied in clinical practice, with the advantages of high resolution, high throughput, and low cost [9, 10].

The detection of CNVs is mainly incidental following invasive procedures conducted due to abnormal ultrasound findings. Because of the risk of fetal loss caused by interventional prenatal diagnostic procedure, most pregnant women with a normal fetal ultrasound preferred prenatal screening to assess the risk of fetal chromosome abnormalities. Maternal serum biochemical markers screening has been used in clinic for several decades, assessing the risk of fetal trisomy 21 and 18 and open neural tube defects. However, the efficiency of this method is not satisfactory. For example, at a false positive rate of 5%, the detection rate of trisomy 21 with the first and second trimester biochemical screening was 82–87% or even lower [11, 12].

Noninvasive prenatal screening (NIPS), based on the analysis discovery of cell free DNA in maternal plasma and the development of NGS technology, has revolutionized the prenatal screening of fetal chromosome abnormalities [13]. NIPS has been recognized as a reliable method to screen trisomy 21, 18 and 13. A recent meta-analysis showed that the detection rate of trisomy 21, 18 and 13 are 99.7%, 97.9% and 99.0%, respectively, and the false positive rate is 0.04% [14]. In addition to its success in detecting common aneuploidies, many literatures reported that extended NIPS has been used with the aim to detect other aneuploidies and CNVs [15–19]. The majority of commercial extended NIPS platforms target common aneuploidies and several common microdeletion/microduplication syndromes (MMS) including 1p36 deletion syndrome, Cri du Chat syndrome, Angelman/ Prader–Willi syndrome, and DiGeorge syndrome [16, 17]. At the same time, some researchers reported that extended NIPS was used to detect both aneuploidies and genome-wide MMS [18, 19]. In December 2022, ACMG strongly recommended that all pregnant women be screened for fetal trisomies 21, 18, 13 and sex chromosome aneuploidies (SCAs) by NIPS. For CNVs, if requested by pregnant women, NIPS can be offered for 22q11.2 deletion syndrome, and it is not recommended to use NIPS for genome-wide CNV screening [2].

For genome-wide CNV screening, many scholars believed that extended NIPS had limited clinical utility, uncertainties regarding positive predictive value (PPV) and negative predictive value (NPV) and the lack of clinical validation of routine use [2, 20]. Meanwhile, there is currently insufficient evidence to support the benefits of NIPS screening for rare autosomal trisomies (RATs). Therefore, more studies are currently needed to help clarify the scope of extended NIPS for CNVs and aneuploids. In addition, considering genetic variation within humans, the frequency and distribution of chromosomal abnormalities may be different among different regions and populations [21]. Here we aim to report the distribution and characteristics of fetal chromosomal abnormalities in Western China to determine the potential scope for extended NIPS.

## Methods

### Participants

Pregnant women who referred for amniocentesis and chromosome testing for clinical indications including advanced age (≥ 35 years), high-risk maternal serum screening, ultrasonographic soft marker detection or voluntary requests between February 2017 and March 2021 were recruited to participate in the study. Those with fetal structural abnormalities detected by ultrasonography were excluded. The clinical study was approved by the Medical Ethics Committee of West China Second University Hospital of Sichuan University (medical research 2016-7). There was no incentive offered for entering the study. Thus, no undue influence on participation existed. All participants gave written informed consents for all investigations, including maternal serum screening, ultrasound scanning, and amniocentesis for detecting fetal chromosomal anomalies.

### Sample preparation and detection

Amniocentesis was performed by needle puncture of the amnion, and 20–25 mL of amniotic fluid was removed by aspiration. Amniocytes were immediately collected by centrifugation, washed thoroughly in phosphate-buffered saline (PBS), and genomic DNA was extracted using the DNeasy blood and tissue kit (Qiagen, Hilden, Germany) according to the manufacturer’s protocol.

All samples were subjected to quantitative fluorescence polymerase chain reaction (QF-PCR) and copy number variation sequencing (CNV-seq). QF-PCR was performed using 21 trisomy/sex chromosome/polyploidy and 18 trisomy/13 trisomy/polyploidy detection kits (DaAn Gene, Guangzhou, China). When QF-PCR results indicated the presence of maternal cells in the samples, CNV-seq and QF-PCR were repeated on the spare samples after cell culture. DNA libraries were prepared using a Chromosome CNV Detection kit (Berry Genomics, Beijing, China) and subsequently sequenced on the Illumina NextSeq500 sequencing platform using a NextSeq500 High Output kit (Illumina, San Diego, CA, USA) according to the manufacturer’s instructions. We compared the reads obtained by NGS with the GRCh37 reference genome and performed bioinformatics analysis to obtain the genomic copy number information of the samples. CNV-seq and QF-PCR were performed according to the manufacturer’s instructions as described previously [22].

For the samples with chromosome abnormalities, other methods were used for verification. Aneuploidy (except trisomy 13, 18, and 21) and all mosaics were verified by karyotyping analysis or fluorescence in situ hybridization. The CNVs identified by CNV-Seq were confirmed by chromosome microarray analysis, multiple ligation probe amplification or repeating the CNV-seq analysis in an independent laboratory. In cases with CNVs findings, CNV-seq was also performed on parental samples to help determine the pathogenicity and inherited patterns of CNVs.

### Analysis and interpretation

**Fig. 1:**
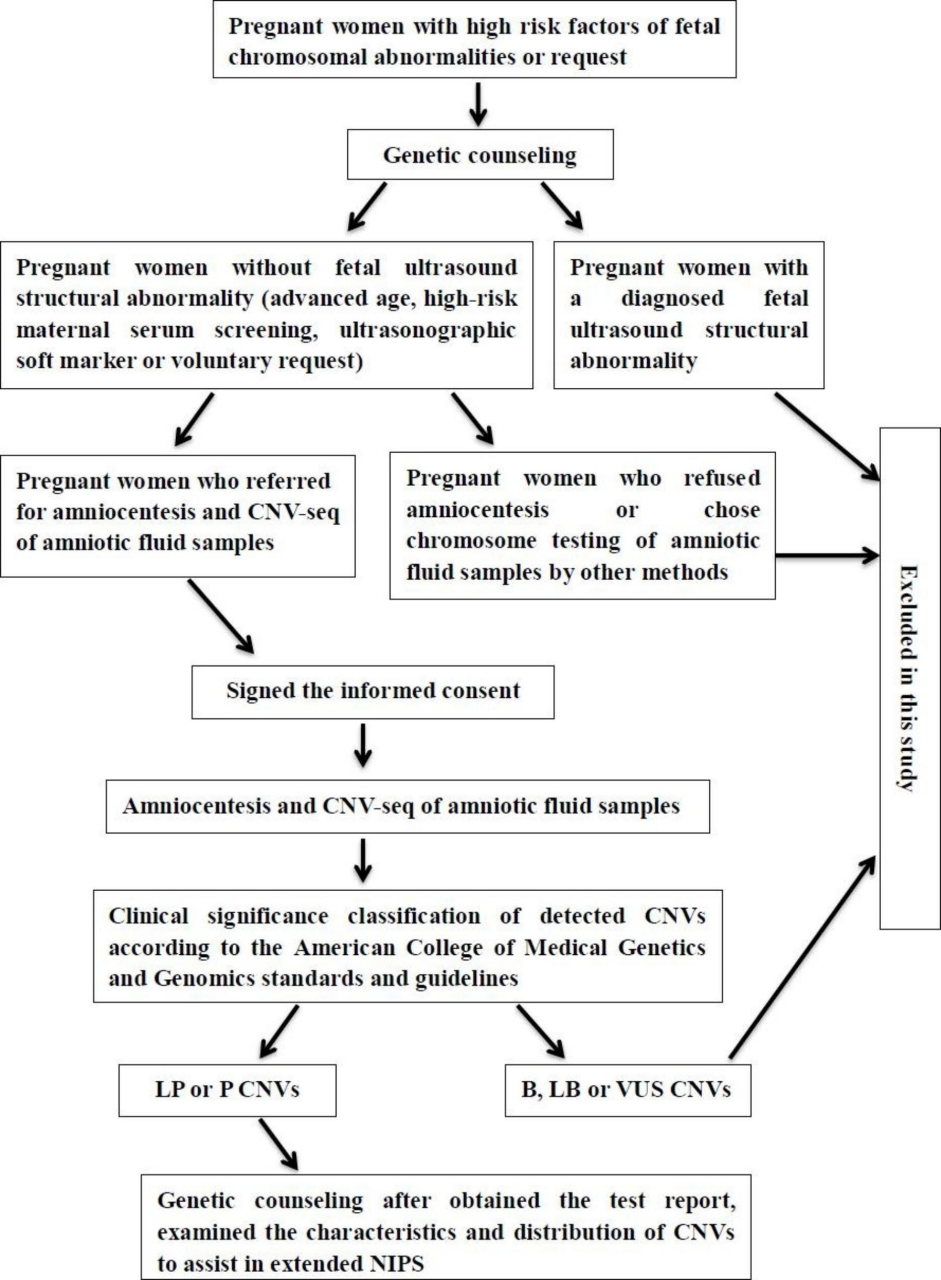
The flowchart of the study design

In this study, the pathogenicity of CNVs > 100 kb was analyzed. The clinical significance of detected CNVs was classified as benign (B), likely benign (LB), variants of uncertain significance (VUS), LP or P according to the American College of Medical Genetics and Genomics standards and guidelines [23]. Detected CNVs were evaluated based on scientific literature reviews and public databases, including ClinGen (http://www.ncbi.nlm.nih.gov/projects/dbvar/clingen/), DECIPHER (http://decipher.sanger.ac.uk/), GeneReviews (http://www.ncbi.nlm.nih.gov/books/NBK1116/), Database of Genomic Variants (DGV, http://dgv.tcag.ca/dgv/app/home/), ClinVar (https://www.ncbi.nlm.nih.gov/clinvar), Genome Aggregation Database (gnomAD, http://gnomad-sg.org/), and Online Mendelian Inheritance in Man (OMIM; http://www.omim.org/). Following classification, we excluded B, LB, and VUS from the study. The flowchart of the study design is shown in Fig. 1.

## Results

**Fig. 2:**
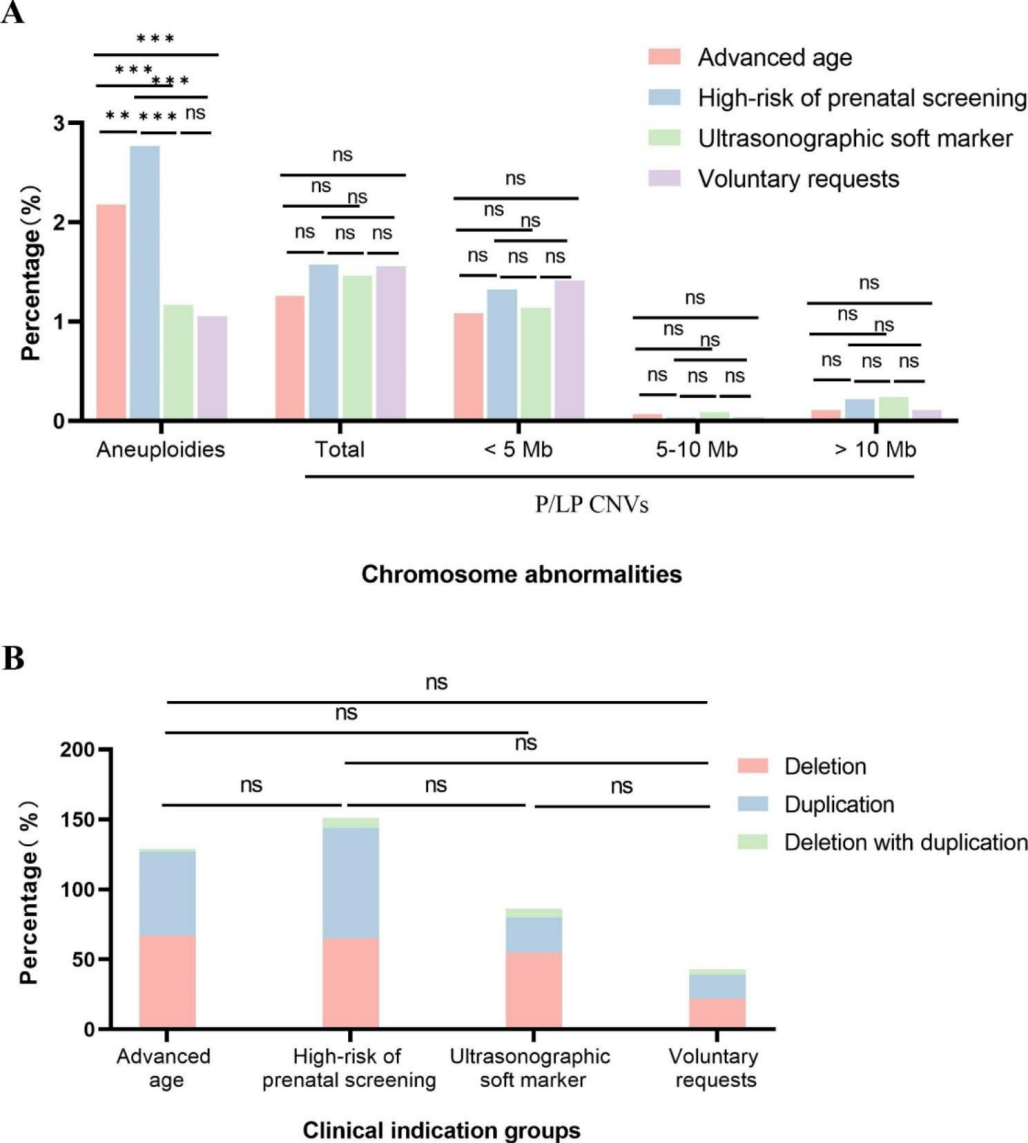
**A.** Chromosome abnormalities in different clinical indication groups. **B.** The CNV characteristics of different clinical indication groups. ns, no significance, P < 0.05; **, P < 0.01; ***, P < 0.001

The final study cohort comprised 28,469 pregnant Chinese women without ultrasonic structural abnormalities, a total of 1,022 with clinically significant fetal chromosome abnormalities were identified, including 587 aneuploidies(2.06%) and 435(1.53%) P/LP CNVs. Advanced age and high-risk of prenatal screening were the most common detection indicators, with higher detection rate of aneuploidies in both groups compared to the other two groups. There was no significant difference in the detection rate of P/LP CNVs among the four clinical indication groups. The incidence of chromosome abnormalities referred by each clinical indication is shown in Table 1; Fig. 2(A).

**Table 1:** Chromosome abnormalities in different clinical indication groups

Group	Sample no.	Average age (years)	Aneuploidies no.(%)	P/LP CNVs			
				no.(%)	< 5 Mb no.(%)	5–10 Mb no.(%)	> 10 Mb no.(%)
**Advanced age**	10,248 (36.00%)	37.16 (34–49)	223 (2.18%)	129 (1.26%)	111 (1.08%)	7 (0.07%)	11 (0.11%)
**High-risk of prenatal screening**	9589 (33.68%)	27.79 (16–34)	266 (2.77%)	151 (1.57%)	127 (1.32%)	3 (0.03%)	21 (0.22%)
**Ultrasonographic soft marker**	5879 (20.65%)	27.64 (17–34)	69 (1.17%)	86 (1.46%)	67 (1.14%)	5 (0.09%)	14 (0.24%)
**Voluntary requests**	2753 (9.67%)	28.60 (16–34)	29 (1.05%)	43 (1.56%)	39 (1.42%)	1 (0.04%)	3 (0.11%)
**Total** **no.(%)**	28,469 (100%)	31.65 (16–49)	587 (2.06%)	409 (1.44%)	344 (1.21%)	16 (0.06%)	49 (0.17%)

Abbreviation: CNVs, copy number variants

### Characteristics of aneuploidies

Of the 28,469 amniotic fluid samples, chromosomal aneuploidies were found in 587 fetuses (587/28,469, 2.06%), all of which were aneuploidies, including 496 non-mosaics (496/587, 84.50%) and 91 mosaics (91/587, 15.50%). Trisomy 21 was the most frequent, followed by SCAs. mosaics were mainly found in the sex chromosomes, as shown in Table 2.

**Table 2:** Distribution of 587 aneuploidies detected in 28,469 fetuses

Group	Non-mosaics		mosaics	
**Chromosomal abnormalities** **no.(%)**	Trisomy 13	10(1.70%)	Trisomy 2/N	1(0.17%)
	Trisomy 18	69(11.75%)	Trisomy 4/N	1(0.17%)
	Trisomy 21	306(52.13%)	Trisomy 9/N	7(1.19%)
	XXY	54(9.20%)	Trisomy 12/N	5(0.85%)
	XYY	18(3.07%)	Trisomy 15/N	1(0.17%)
	XXX	35(5.96%)	Trisomy 16/N	1(0.17%)
	XXXX	1(0.17%)	Trisomy 21/N	16(2.73%)
	XO	3(0.51%)	Trisomy 22/N	1(0.17%)
	-	-	sex chromosome	58(9.88%)
	total	496(84.50%)	total	91(15.50%)

Note: Data are presented as numbers and percentages for every group

Abbreviation: N, normal

### Characteristics of P/LP CNVs

**Fig. 3:**
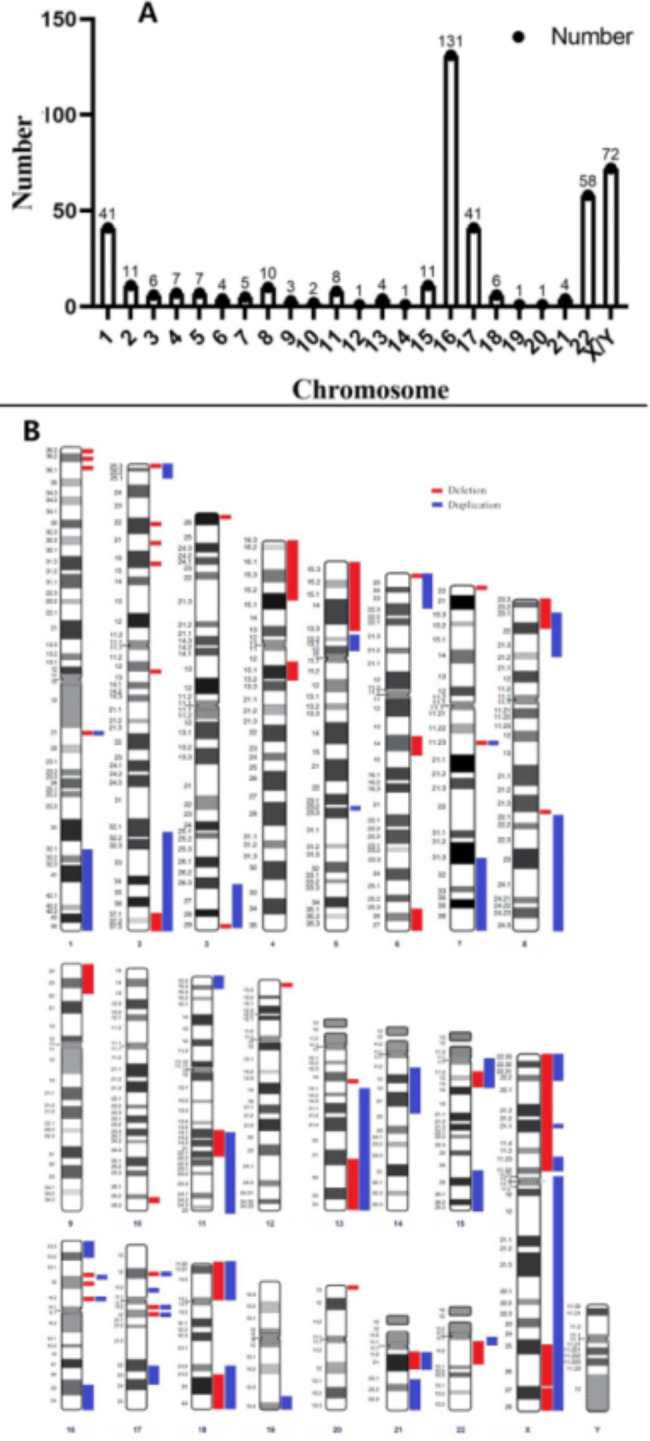
**A.** The chromosome distribution of P/LP CNVs detected; **B.** Chromosome regional distribution of P/LP CNVs

A total of 435 P/LP CNVs were found in 409 fetuses (409/28,469, 1.44%), including 231 deletions (231/435, 53.10%) and 204 duplications (204/435, 46.90%). Deletions were observed in 209 cases (209/28,469, 0.73%) and duplications were observed in 181 cases (181/28,469, 0.64%). In 19 cases (19/28,469, 0.07%), deletion and duplication were both found. P/LP CNVs were found in all chromosomes. P/LP CNVs in chromosomes 16, 22, and X exhibited the highest frequencies. Figure 3 shows the specific distribution of P/LP CNVs on each chromosome. Of the 435 pathogenic or likely pathogenic CNVs detected, 330 cases of MMS were associated with classic chromosome diseases. A total of 38 syndromes were detected in 14 chromosomes, and the three most frequently detected were 16p13.11 recurrent microduplication (60/330), 22q11 duplication syndrome (36/330), and steroid syndrome deficiency (31/330) (Supplementary Table 1).

**Fig. 4:**
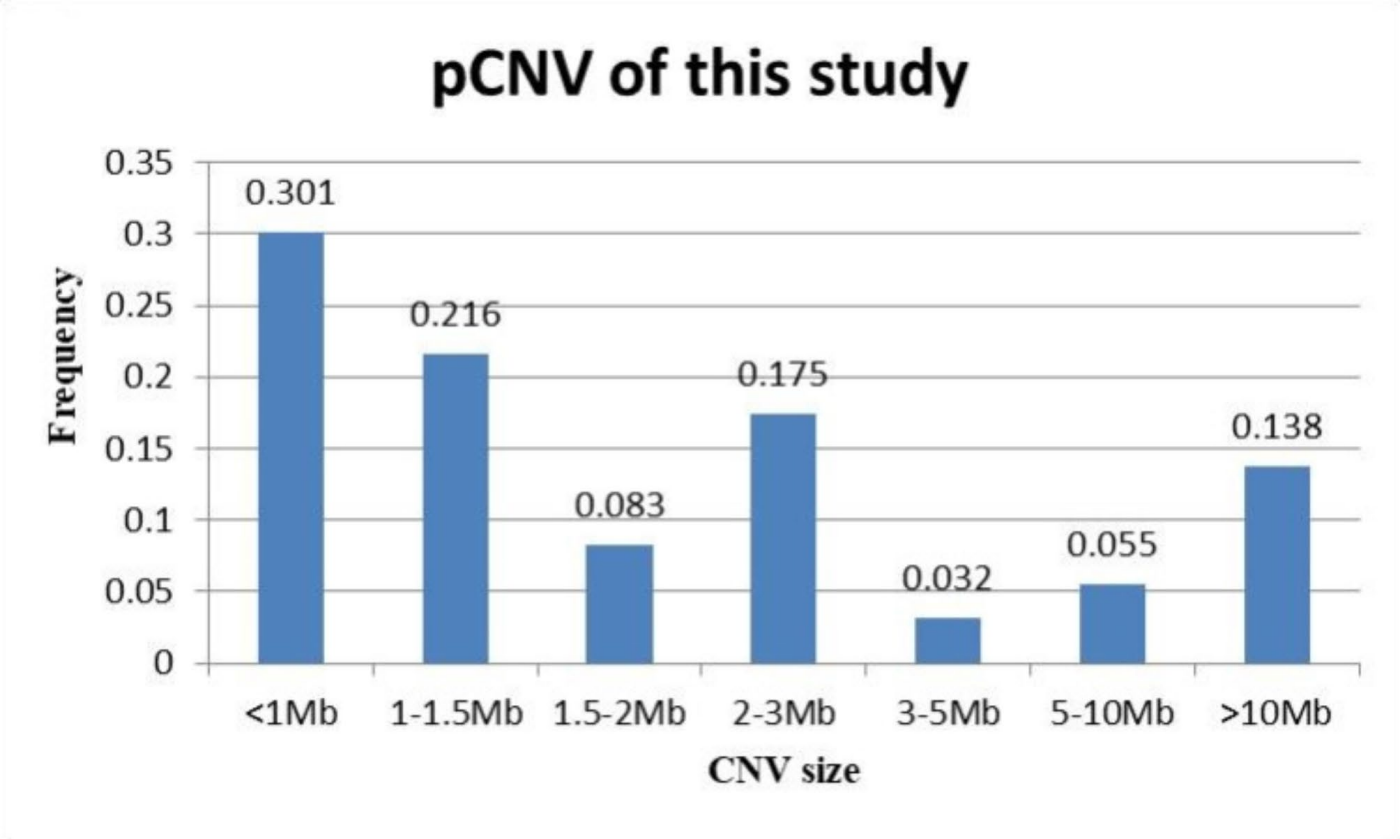
Size distributions of pathogenic/likely pathogenic copy number variations (P/LP CNVs)

Most P/LP CNVs (351/435, 80.69%) were < 5 Mb. 24/435 (5.52%) were between 5 and 10 Mb and 60/435 (13.79%) were > 10 Mb. There was no significant difference in the composition ratio of deletion, duplication, and deletion with duplication CNVs among different clinical indication groups. The relevant information is shown in Table 3; Fig. 2(B). Of the 435 P/LP CNVs, 337 CNVs (337/435, 77.47%) were smaller than 3 Mb. The size distribution of P/LP CNVs detected in this study is shown in Fig. 4.

**Table 3:** Characteristics of P/LP CNVs in different clinical indication groups

P/LP CNVs	Group, no.(%)				
	Advanced age	High-risk of prenatal screening	Ultrasonographic soft marker	Voluntary requests	Total samples
**Deletion**	67(51.94%)	65(43.05%)	55(63.95%)	22(51.16%)	209(51.10%)
**Duplication**	60(46.51%)	79(52.32%)	25(29.07%)	17(39.53%)	181(44.25%)
**Deletion with duplication**	2(1.55%)	7(4.64%)	6(6.98%)	4(9.3%)	19(4.65%)
**Total no.(%)**	129(31.54%)	151(36.92%)	86(21.03%)	43(10.51%)	409(100%)

Abbreviation: CNVs, copy number variants

## Discussion

In this large prospective study investigating the distribution and characteristics of pathogenic chromosomal variations in prenatal diagnosis samples to explore the target scope of extended NIPS, aneuploidies were more common than P/LP CNVs in fetuses without ultrasonic structural abnormalities. P/LP CNVs were seen in all chromosomes, but with distribution skewed towards some specific regions such as distal part of the chromosomes and low copy repeat regions. The majority of P/LP CNVs were less than 3 Mb, which is below the resolution of most extended NIPS platforms, indicating that the scope should be reconsidered.

In humans, aneuploidies are common and originate from either meiotic nondisjunction errors or mitotic replication errors, often in the preimplantation embryo stage [24]. In this study, aneuploidies were identified in 2.06% (587/28,469) of fetuses, of which trisomy 21 was the most common, followed by SCAs. The chromosomal abnormalities of trisomy 13, 18, and 21 have been traditional targets of NIPS in China. The results showed SCAs are quite common (169/587, 28.79%), which supports that sex chromosomes could be included in the routine scope of extended NIPS. It is worth mentioning that the suggestive method of sex chromosome screening results should consider avoiding the risk of sex selection. Although the ACMG guidelines recommend routine screening for SCAs, clinical experience has demonstrated that not all pregnant women will pursue screening for SCAs, and laboratories offering NIPS generally provide an opt-out option [2]. Except 13, 18, 21, and sex chromosomes, aneuploidies of other chromosomes were mosaics, which the incidence was 0.06% (17/28,469). An explanation for this might be that most aneuploidies result in either embryo implantation failure, growth arrest, or early miscarriage during the first trimester [25]. Based on the results, we consider that other chromosomes can be excluded from the routine target scope of extended NIPS for its expected low operational benefit.

In the past, people mainly focused on the rate of P/LP CNVs in fetuses with ultrasonic structural abnormalities [26]. Our data showed that fetuses met a 1.44% chance of P/LP CNVs even without ultrasonic abnormalities. Previous study suggested that P/LP CNVs account for more than 2/3 of chromosome aberrations which have historically accounted for more than 80% of genetic birth defects [6]. Meanwhile, P/LP CNVs is one of the most common causes in birth defects, second only to structural malformations [27]. The actual detection of P/LP CNVs mainly depended on incidental discovery during prenatal diagnosis. However, due to the well performance of NIPS in aneuploidies screening, its application may reduce the rate of invasive prenatal diagnosis [28, 29]. Therefore, the probability of prenatal ‘accidental’ detection of P/LP CNVs estimates reduced, resulting in the increase of birth defects caused by P/LP CNVs. Extended NIPS is applied for P/LP CNVs screening seems a good way to solve the problem, but the scope is difficult to determine. Our results suggested that the distribution of P/LP CNVs in the genome is not uniform, although they were found in all chromosomes. P/LP CNVs were most common on distal ends of the chromosomes, and on chromosome low copy repeat regions (16p13.11, 22q11.2, 1q21.1, and 17p12) [30]. Therefore, we suggest that the extended NIPS scope for CNVs should focus on these regions. Meanwhile, many recurrent MMS were found in these susceptible loci, accounting for 40.69% of the total P/LP CNVs. The reasons for unequilibrium distribution of CNVs are complex and diverse, and may be related to the regional characteristics of chromosomes and specific lineage selection pressure. The human genome contains a wide range of repetitive sequences, and these unstable repetitive sequences lead to rearrangements within or between chromosomes during meiosis, thus generating CNVs [31]. The ends of chromosomes and low copy repeat regions cover lots of repetitive sequences, leading the instability increased, so it is easier to generate CNVs in these regions. Meanwhile, some studies have shown that the lineage distribution of CNVs is affected by selective pressures. The distribution of these CNVs may be the result of selection under pressure [32]. Among 330 cases of MMS, 16p13.11 recurrent microduplication was the most common, accounting for 18.18% (60/330). The short arm of Chromosome 16 is rich in repetitive sequences, including more than 10% of its euchromatin. Therefore, Chromosome 16 is the hot spot of replication errors in the human genome, which eventually leads to the occurrence of many MMS, especially in the 16p13.11 region [33]. The clinical phenotype of 16p13.11 recurrent microduplication varies greatly, which can be manifested as autism spectrum disorder, learning difculties, brain MRI abnormalities, heart malformation and other abnormalities. The penetrance was approximately 7–8%, and about 80% of the cases are inherited from the father or mother with normal phenotype [33–35]. Therefore, this poses challenges for prenatal counseling because the associated neurodevelopmental phenotypes cannot be ascertained prenatally and it is difficult to quantify the risk to the fetus. Therefore, if the results of NIPS indicates that the fetus may have recurrent CNVs, clinicians should tell the pregnant women in detail about the PPV of NIPS, phenotypic characteristics, penetrance, and origin of the CNV. It is up to the pregnant women and their families to decide whether to receive interventional prenatal diagnosis.

Actually, CNVs can occur in any pregnancy independent of maternal age. Therefore, the study of spectrum and characteristics of fetal chromosome abnormalities is of great value in determining the scope and strategy of extended NIPS. The frequency and distribution of chromosomal abnormalities may be different among different regions and populations [21]. For pregnant women in Hong Kong, 375 of 23,865 fetuses (1.6%) carried P CNVs for any indication for invasive testing. A total of 428 P CNVs were detected in these fetuses, of which 280 (65.42%) were deletions and 148 (34.58%) were duplications. 84.1% were less than 5 Mb in size. The research results provided valuable data for extended NIPS among pregnant women in Hong Kong [36]. In our study, P/LP CNVs were found in 409 fetuses (409/28,469, 1.44%), and 80.69% P/LP CNVs were < 5 Mb. Compared to Chau’s research data, the detection rate of P/LP CNVs in our study is lower, presumably because their study samples included fetuses with abnormal ultrasound findings [36]. Several studies have explored the application of extended NIPS for CNVs [16–19, 37–40]. Hyblova et al. showed that the sensitivity of extended NIPS they used for CNVs > 3 Mb is 100%, but there are still challenges detecting CNVs < 3 Mb [41]. Another study showed that extended NIPS could detect 83% of CNVs > 6 Mb, but only 20% of CNVs were < 6 Mb [42]. Similarly, another study found that 90.9% of CNVs > 5 Mb could be detected by extended NIPS, but only 14.3% of CNVs < 5 Mb could be found [38]. Actually,, the size of most P/LP CNVs (77.47%) found in this study was < 3 Mb. According to our findings, the size of most P/LP CNVs is beyond the detection limit of many extended NIPS platforms. It means that under the traditional methods and strategies at present, most P/LP CNVs will be missed. It is worth noting that some studies have shown that the SNP-based NIPS has advantages and high sensitivity in detecting MMS in some regions (such as 22q11.2), which could be used by extended NIPS for P/LP CNVs screening [40, 43, 44]. However, a systematic review showed that the PPV of SNP-based extended NIPS for MMS was approximately 44.1% (95%CI = 31.49–63.07) [45]. Currently, even with the use of genome-wide NIPS, there is still approximately 54.1% of clinically significant CNVs that found by prenatal invasive testing being missed [20]. In conclusion, based on the existing platform for extended NIPS, the screening effect of P/LP CNVs seems to be unsatisfactory.

Based on our research findings, for the pregnant women in Western China, because most P/LP CNVs were less than 3 Mb, it is recommended to optimize data analysis for the coverage area of P/LP CNVs, especially for the high-frequency areas. At the same time, increase the density of capture probes in the target area or increase the read length and depth of sequencing to discover as many P/LP CNVs as possible. In addition, sufficient preclinical validation is still needed to ensure the clinical effect of extended NIPS. The sample size of this study is large, but the samples were from pregnant women in Western China. Due to wide geographical location, large population and diverse ethnic groups, more research is needed to determine whether the data in this study represent the CNV characteristics of fetuses with normal ultrasound scans in China. We hope to obtain samples nationwide in the future to clarify the CNV characteristics of more populations and provide the theoretical basis for prenatal screening of CNVs.

## Conclusions

For fetuses with normal ultrasound scans in Western China, aneuploidies were identified in 2.06% of fetuses, of which trisomy 21 was the most common, followed by SCAs. P/LP CNVs were found in 1.44% of fetuses, located on all chromosomes, and the size of most P/LP CNVs (77.47%) was less than 3 Mb. The scope of extended NIPS should include common aneuploidies and high-frequency CNVs as much as possible, and sufficient preclinical validation is still needed to ensure the clinical effect of extended NIPS.

### Electronic supplementary material

Below is the link to the electronic supplementary material.

Supplementary Material 1

## Data Availability

The datasets generated and/or analyzed during the current study are not publicly available due to upload failure caused by the oversized but are available from the corresponding author on reasonable request.

## List of abbreviations

NIPS: noninvasive prenatal screening
CNVs: copy number variants
CNV-seq: copy number variation sequencing
P/ LP: pathogenic/ likely pathogenic
MMS: microdeletion/microduplication syndromes
SCAs: sex chromosome aneuploidies
PPV: positive predictive value
NPV: negative predictive value
RATs: rare autosomal trisomies
