# Supplementary material for: Defining the scope of extended NIPS in Western China: evidence from a large cohort of fetuses with normal ultrasound scans
Source: BMC Pregnancy Childbirth. 2023 Aug 19;23:593. doi: 10.1186/s12884-023-05921-x (PMC10439619; doi:10.1186/s12884-023-05921-x)
Supplement: Supplementary file 1 — Supplementary Material 1 [file 12884_2023_5921_MOESM1_ESM.docx]

| **Supplementary Table 1. Microdeletion/Microduplication syndromes detected in 28469 fetuses.** | | |
| --- | --- | --- |
| **Chromosome** | **Microdeletion/Microduplication syndromes** | **Cases** |
| 1 | 1q21.1 recurrent microdeletion (susceptibility locus for neurodevelopmental disorders) | 12 |
|  | 1q21.1 recurrent microduplication (possible susceptibility locus for neurodevelopmental disorders) | 9 |
|  | 1q21.1 susceptibility locus for Thrombocytopenia-Absent Radius (TAR) syndrome | 15 |
| 2 | 2p21 Microdeletion Syndrome | 1 |
|  | 2q37 monosomy | 2 |
| 3 | 3q29 microdeletion syndrome | 3 |
|  | 3q29 microduplication syndrome | 2 |
| 4 | Wolf-Hirschhorn Syndrome | 5 |
| 5 | Adult-onset autosomal dominant leukodystrophy (ADLD) | 1 |
|  | Cri du Chat Syndrome (5p deletion) | 4 |
| 7 | 7q11.23 duplication syndrome | 1 |
|  | Williams-Beuren Syndrome (WBS) | 1 |
| 8 | 8p23.1 deletion syndrome | 2 |
|  | 8p23.1 duplication syndrome | 1 |
| 12 | 12p13.33 Microdeletion Syndrome | 1 |
| 15 | 15q Duplication Syndrome and Related Disorders | 1 |
|  | 15q13.3 microdeletion syndrome | 5 |
|  | 15q26 overgrowth syndrome | 2 |
|  | Angelman syndrome/Prader-Willi syndrome | 1 |
| 16 | 16p11.2 microduplication syndrome | 24 |
|  | 16p11.2 Recurrent Microdeletion | 11 |
|  | 16p13.11 recurrent microdeletion (neurocognitive disorder susceptibility locus) | 19 |
|  | 16p13.11 recurrent microduplication (neurocognitive disorder susceptibility locus) | 60 |
|  | Recurrent 16p12.1 microdeletion (neurodevelopmental susceptibility locus) | 9 |
| 17 | 17q12 Recurrent Duplication | 6 |
|  | Charcot-Marie-Tooth syndrome type 1A (CMT1A) | 11 |
|  | Hereditary Liability to Pressure Palsies (HNPP) | 11 |
|  | NF1-microdeletion syndrome | 2 |
|  | Potocki-Lupski syndrome (17p11.2 duplication syndrome) | 2 |
|  | RCAD (renal cysts and diabetes) | 8 |
| 21 | Early-onset Alzheimer disease with cerebral amyloid angiopathy | 1 |
| 22 | 22q11 deletion syndrome (Velocardiofacial / DiGeorge syndrome) | 15 |
|  | 22q11 duplication syndrome | 36 |
|  | 22q11.2 distal deletion syndrome | 5 |
| X | Steroid sulphatase deficiency (STS) | 31 |
|  | Xp11.22-linked intellectual disability | 2 |
|  | Xp11.22-p11.23 Microduplication | 1 |
|  | Xq28 Duplication Syndrome,Int22h1/ Int22h2 Mediated | 7 |
